# Dr. Alfred M. Gamman

**Published:** 1917-12

**Authors:** 


**Dr. Alfred M. Gamman,** N. Y. Homoeopathic 1866, of Corning, N. Y., died in Kansas City, Oct. 5, aged 65, (sic, Jour. A. M. A., obviously incompatible with date of graduation), of nephritis. He was a pioneer oil man of Tulsa, Ok.

